# Hsa-miR-133a-3p, miR-1-3p, GOLPH3 and JUP combination results in a good biomarker to distinguish between prostate cancer and non-prostate cancer patients

**DOI:** 10.3389/fonc.2022.997457

**Published:** 2022-10-26

**Authors:** Rocío Belén Duca, Cintia Massillo, Paula Lucía Farré, Karen Daniela Graña, Juana Moro, Kevin Gardner, Ezequiel Lacunza, Adriana De Siervi

**Affiliations:** ^1^ Laboratorio de Oncología Molecular y Nuevos Blancos Terapéuticos, Instituto de Biología y Medicina Experimental (IBYME), Consejo Nacional de Investigaciones Científicas y Técnicas (CONICET), Buenos Aires, Argentina; ^2^ Department of Pathology and Cell Biology, Columbia University Medical Center, New York, NY, United States; ^3^ Centro de Investigaciones Inmunológicas Básicas y Aplicadas (CINIBA), Facultad de Ciencias Médicas, Universidad Nacional de La Plata, Buenos Aires, Argentina

**Keywords:** prostate cancer, microRNA, high fat diet, methylation, oncogenes

## Abstract

The incidence and mortality of Prostate Cancer (PCa) worldwide correlate with age and bad dietary habits. Previously, we investigated the mRNA/miRNA role on PCa development and progression using high fat diet (HFD) fed mice. Here our main goal was to investigate the effect of HFD on the expression of PCa-related miRNAs and their relevance in PCa patients. We identified 6 up- and 18 down-regulated miRNAs in TRAMP-C1 mice prostate tumors under HFD conditions using miRNA microarrays. Three down-regulated miRNAs: mmu-miR-133a-3p, -1a-3p and -29c-3p were validated in TRAMP-C1 mice prostate tumor by stem-loop RT-qPCR. Hsa-miR-133a-3p/1-3p expression levels were significantly decreased in PCa compared to normal tissues while hsa-miR-133a-3p was found to be further decreased in metastatic prostate cancer tumors compared to non-metastatic PCa. We examined the promoter region of hsa-miR-133a-3p/1-3p genes and compared methylation at these loci with mature miRNA expression. We found that hsa-miR-1-2/miR-133a-1 cluster promoter hypermethylation decreased hsa-miR-133a-3p/1-3p expression in PCa. GOLPH3 and JUP, two hsa-miR-133a-3p and miR-1-3p predicted target genes, were up-regulated in PCa. ROC analysis showed that the combination of hsa-miR-133a-3p, miR-1-3p, GOLPH3 and JUP is a promising panel biomarker to distinguish between PCa and normal adjacent tissue (NAT). These results link PCa aggressiveness to the attenuation of hsa-miR-133a-3p and miR-1-3p expression by promoter hypermethylation. Hsa-miR-133a-3p and miR-1-3p down-regulation may enhance PCa aggressiveness in part by targeting GOLPH3 and JUP.

## Introduction

Prostate cancer (PCa) is currently the most commonly diagnosed type of cancer and the fifth leading cause of cancer deaths among men over the age of 50 years worldwide (https://gco.iarc.fr/). Although PCa is a multifactorial disease, different epidemiological studies suggested that lifestyle and environmental factors influence the development and progression of this disease (1). Dietary fats and obesity have the potential to cause PCa initiation, promotion and progression (2). The proposed mechanisms for PCa induced by dietary fats are divided into growth factor signaling, lipid metabolism, inflammation and hormonal modulation among others (2). However, the underlying molecular mechanisms responsible for the effect of high fat diet (HFD) on PCa development and progression remain unknown.

Previously, we generated several preclinical mice models to investigate the impact of HFD on PCa development and progression. We reported that C-terminal binding protein 1 (CTBP1) depletion in androgen-insensitive PCa xenografts from HFD-fed mice modulated the expression of mRNAs and microRNAs (miRNAs) involved in cancer related pathways which impacts on PCa proliferation and invasion (3–5). Additionally, recent androgen-sensitive PCa allografts and HFD mice model demonstrated that high fat intake significantly increased tumor growth. Tumors developed in HFD fed mice showed overexpression of oncogenes and oncomiRs compared to control diet (CD) (6–8).

MiRNAs are endogenous small non-coding RNAs (18-22 nucleotides) that regulate gene expression. Compelling evidence have demonstrated that miRNA expression is deregulated in several human cancer types through numerous mechanisms, including amplification or deletion of miRNA genes, abnormal miRNAs transcription, deregulated epigenetic changes and defects in the miRNA biogenesis machinery (9). MiRNAs can function either as oncogenes (oncomiRs) or tumor suppressors (tsmiRs) under certain conditions. The deregulated miRNAs have been shown to affect several hallmarks of cancer, including sustaining proliferative signaling, evading growth suppressors, resisting cell death, activating invasion and metastasis, and inducing angiogenesis (9). In PCa, several miRNAs have been proposed to regulate cell proliferation, cell cycle, apoptosis, as well as invasion and adhesion processes (9). In addition, diet and lifestyle factors are involved in the regulation of miRNA expression in different tissues and pathologies, including cancer (10–12). Finally, an increasing number of studies have identified miRNAs as potential biomarkers for PCa diagnosis, prognosis and therapy.

Here our main goal was to investigate the effect of HFD on the expression of cancer-related miRNAs in prostate tumors and their relevance in PCa patients. Starting from microarray data from prostate tumors obtained from CD or HFD fed mice we focused on the role of two miRNAs (miR-133a-3p/1-3p) and their target genes. Using PCa patient samples from public databases, we examined the expression levels of hsa-miR-133a-3p/1-3p. The biological roles of hsa-miR-133a-3p/1-3p and their relevant target genes were investigated by bioinformatics approaches. Finally, the promoter methylation of hsa-miR-133a-3p/1-3p host genes and their correlation with mature miRNA expression was evaluated.

**Figure 1:**
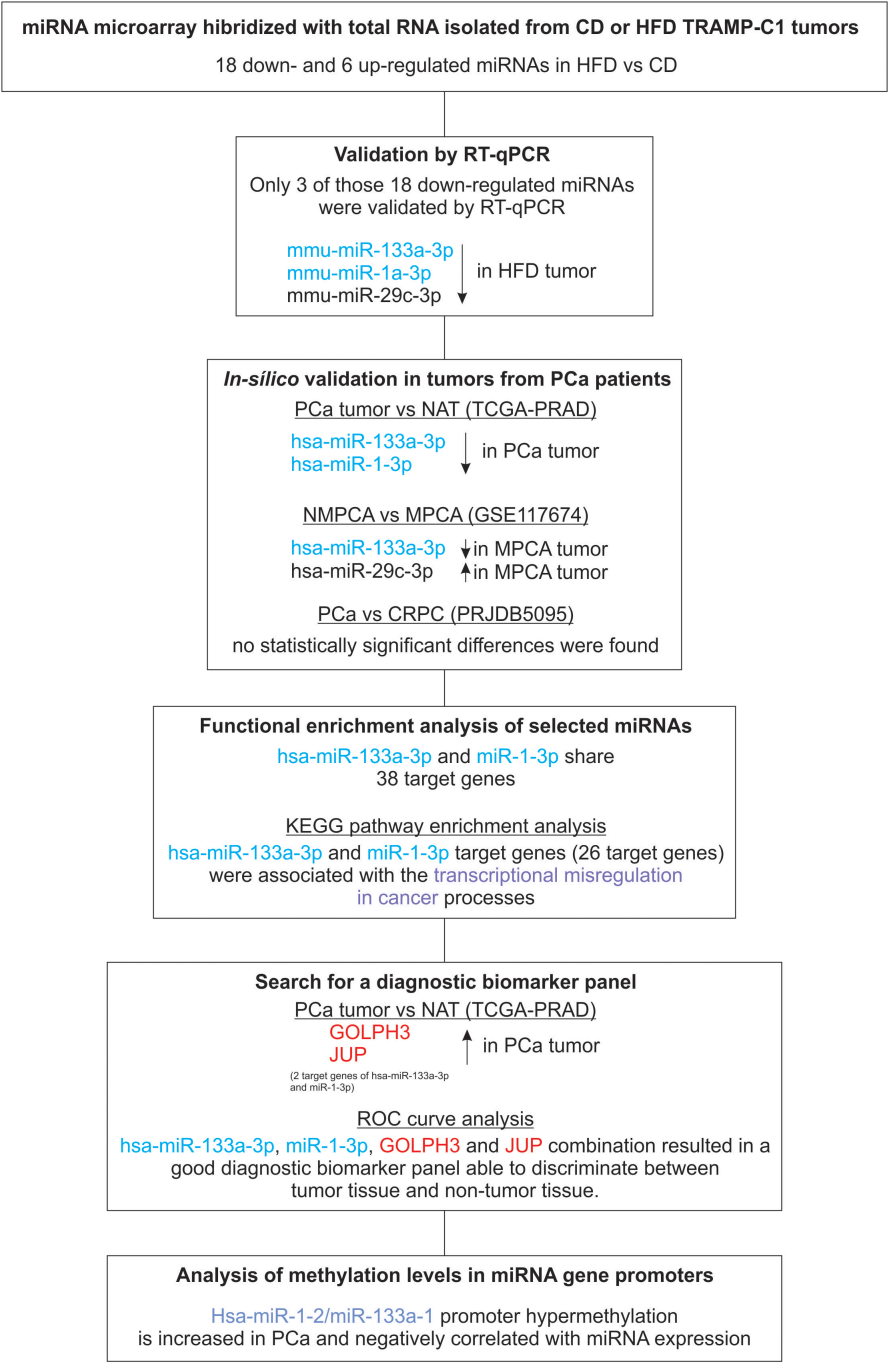
Methodology flow chart. Selected miRNAs and target genes are indicated in blue and red, respectively.

**Figure 1** summarizes the steps of the methodology we followed in this study.

## Materials and methods

### Cell culture

TRAMP-C1 cell line (ATCC: CRL-2730, Manassas, VA, USA) was cultured in DMEM medium (GIBCO, Thermo Scientific, Massachusetts, USA) supplemented with 10% of fetal bovine serum, antibiotics and 0.25 IU/μl of human recombinant insulin in a 5% CO_2_ humidified incubator at 37°C.

### PCa allograft and HFD murine model

Six-weeks-old C57BL/6J male mice (N=12) were housed under pathogen-free conditions following the IBYME’s animal care guidelines. Mice were randomized into two dietary groups and fed ad libitum during 20 weeks with CD (3,120 kcal/kg, 5% fat) or HFD (4,520 kcal/kg, 37% fat). After 12 weeks of diet, 5 × 10_6_ TRAMP-C1 cells were subcutaneously injected. Animals were sacrificed in the 20th week and tumor samples were collected. The metabolic state of the animals and tumor volume were analyzed as previously described (6).

### RNA isolation and microarrays analysis

For microarray analysis, total RNA from CD or HFD allografts was isolated using TriReagent (Molecular Research Center) and hybridized with GeneChip^®^ miRNA 4.0 Array (Affymetrix) (N=3 per group). For miRNA expression analysis, we employed the Limma and pd.mirna.4.0 packages in the R/Bioconductor environment. For differential expression analysis we used the Rank Product Method for two class unpaired data and a fold discovery rate (FDR) < 0.05 (13).

### RNA isolation and stem-loop RT-qPCR

Total RNA from allografts and plasma was isolated using TriReagent (Molecular Research Center). For plasma samples, cel-miR-39 synthetic miRNA (20 fmol) was spiked in before RNA isolation. miRNAs were retrotranscribed using the stem-loop method as previously described (4, 14, 15). Briefly, 100 ng from allografts or 4 μl of total RNA from plasma and 0.07 μM of stem-loop primer were preheated (70°C, 5min). Retrotranscription (RT) was performed using M-MLV reverse transcriptase (Promega) and incubated in MyGenie96 Thermal Block (Bioneer) (30min 16°C, 60min 42°C, 2min 70°C). qPCRs were run in 10 μl with 0.1 μM of each primer and 5 μl of PowerUp™ SYBR™ Green Master Mix (Thermo Fisher), in StepOne Plus Real Time PCR (Applied Biosystems) (50°C 2min, 95°C 10 min, 40 cycles: 95°C 15s, annealing temperature 15s, 60°C 1min and 95°C 15s) as previously described (7). All reactions were run in duplicate. The expression levels of miRNAs were calculated using ΔΔCT method normalizing to hsa-miR-103a-3p and hsa-miR-191-5p levels and control. The expression levels of plasma miRNAs were normalized to cel-miR-39. Statistical analysis was performed using Mann-Whitney test. Primer sequences for miRNA stem-loop RT-qPCR are listed in **Table S2**.

### Bioinformatic analysis databases

MiRNA and gene expression of prostate tumors of patients was obtained from TCGA Prostate Cancer (PRAD) cohort available in the UCSC Xena resource (16) (https://xenabrowser.net/). PCa samples (n=497) and normal adjacent tissue (NAT) (n=52) with expression data of miRNA mature strand [miRNA-Seq (IlluminaHiSeq_miRNASeq)] and genes [RNAseq (IlluminaHiSeq)] as log2 (RPM+1) values were included in the present study. Additionally, miRNA expression profile in metastatic PCa patients was analyzed in a cohort of 19 patients with metastases diagnosis after radical prostatectomy (MPCA (metastatic prostate cancer)) and 19 patients without evidence of disease recurrence (NMPCA (non-metastatic prostate cancer)), using next generation whole miRNome sequencing results from Nam’s work available from GEO dataset (GSE117674) (17). GSE117674 was generated by Ion Torrent S5 XL (Homo sapiens) high-throughput sequencing. MiRNA expression levels in tumors of patients with PCa and castrate-resistant prostate cancer (CRPC) were obtained from the Sequence Read Archive (SRA) data (PRJDB5095) available in miTED bioinformatics tool (https://dianalab.e-ce.uth.gr/mited/#/expressions). Three PCa and three CRPC samples were included in the present study, whose data was downloaded as log2 (RPM) values. Finally, circulating miRNA expression profile was analyzed in the peripheral blood of a cohort of patients with type 2 diabetes (n=6) and healthy donors (HD) (n=4) using microarrays data available from GSE27645. Normalized signal intensity data was downloaded from the Gene Expression Omnibus (GEO) public functional genomics data repository (https://www.ncbi.nlm.nih.gov/geo/). GSE27645 was generated by miRCURY LNA microRNA Array, v.11.0.

Data normalization and homogeneity of variances was assessed using Shapiro–Wilk test, and boxplot or Levene test, respectively. To compare the expression between PCa and NAT samples (N=52 per group) Paired Sample t-test or Sign Median test (using the signmedian.test R package) were applied. Also, Student’s t test or Mann-Whitney test were used to analyze the statistical differences between NMPCA and MPCA (n=19 per group), and between PCa and CRPC (n=3 per group).

### Principal component analysis

Predicted target genes of hsa-miR-133a-3p and hsa-miR-1-3p were obtained from microT-CDS resource. To identify common target genes, we used Venn diagrams (http://bioinformatics.psb.ugent.be/webtools/Venn/).

To the obtained gene list, publicly available log2(norm_count+1) gene expression values for TCGA Prostate Cancer (PRAD) and the GTEx project patient samples were downloaded from UCSC’s Xena Browser (http://xena.ucsc.edu/) (16). PCA was performed to determine samples distribution based on the expression of the target genes identified from the downregulated miRNAs. We included in the analysis normal prostates (n=100) (GTEx), normal adjacent tissue (NAT) (n=52) and prostate tumors (n=495) (TCGA-PRAD) samples. For PCA plots, the R function “prcomp” from stats package (version 4.0.2) was used.

### Functional enrichment analysis

To investigate the functional role of selected miRNAs, we performed Kyoto Encyclopedia of Genes and Genomes (KEGG) pathway analysis and Gene Ontology (GO) annotation using DIANA-miRPath v3 tool (http://snf-515788.vm.okeanos.grnet.gr/), from a list of predicted target genes derived from microT-CDS. The top 10 of the statistically significant terms (p-Value <0.05) were selected. For dot plot, ggplot2 package was used.

### Correlation matrix

Expression levels of selected miRNAs and target genes were obtained from TCGA-PRAD patient cohort data available in UCSC Xena. Expression data of miRNA mature strand [miRNA-Seq (IlluminaHiSeq_miRNASeq)] and genes [RNAseq (IlluminaHiSeq)] were downloaded as log2 (RPM+1) values. PCa samples from 495 patients were included in the present analysis. We generated a correlation matrix between hsa-miR-133a-3p, hsa-miR-1-3p and the selected target genes, applying the Spearman correlation coefficient using the Hmisc R package. For the selected miRNAs, target genes with a negative correlation coefficient rho plus p-Value <0.05 were selected for further analysis. Also, for correlation matrix graphical representation, the R function “chart.Correlation” from PerformanceAnalytics package (version 4.0.2) was used.

### Receiver-operating characteristic analysis

To evaluate the power of hsa-miR-133a-3p, miR-1-3p, GOLPH3 and JUP to distinguish between tumor and NAT, we performed a receiver-operating characteristic (ROC) analysis using the expression data available in the TCGA-PRAD dataset. The ROC curve, the area under the curve (AUC), sensitivity, specificity and the optimal point were plotted and calculated for the miRNAs, genes and their combination using the R function “roc” from pROC package (version 4.0.5)

### Integrated mature miRNA expression and promoter methylation analysis

Hsa-miR-1-2/miR-133a-1 and miR-1-1/miR-133a-2 promoter methylation was evaluated in 52 paired PCa and NAT samples from the TCGA-PRAD cohort. DNA methylation data (Illumina Infinium HumanMethylation450) was downloaded as beta value using UCSC Xena resource. The Ensembl browser (http://www.ensembl.org) was employed to identify the coding genes of the mentioned miRNAs: MIR1-2: chr18:19,408,965-19,409,049, MIR133A1: chr18:19,405,659-19,405,746, MIR1-1: chr20:62,554,306-62,554,376, MIR133A2: chr20:62,564,912-62,565,013. Methylation probes targeting miRNA promoter regions were identified by mapping 5000 base pairs upstream of miRNA TSS (hsa-miR-1-2/miR-133a-1 promoter: chr18:19,409,049-19,414,049, hsa-miR-1-1/miR-133a-2 promoter: chr20:62,554,306-5000-62,544,306). The correlation between promoter methylation and mature miRNA expression was assessed by a Spearman correlation analysis in 497 PCa samples using GraphPad Prism 8.0.1.

## Results

### HFD induces miRNA expression in TRAMP-C1 derived tumors

**Figure 2:**
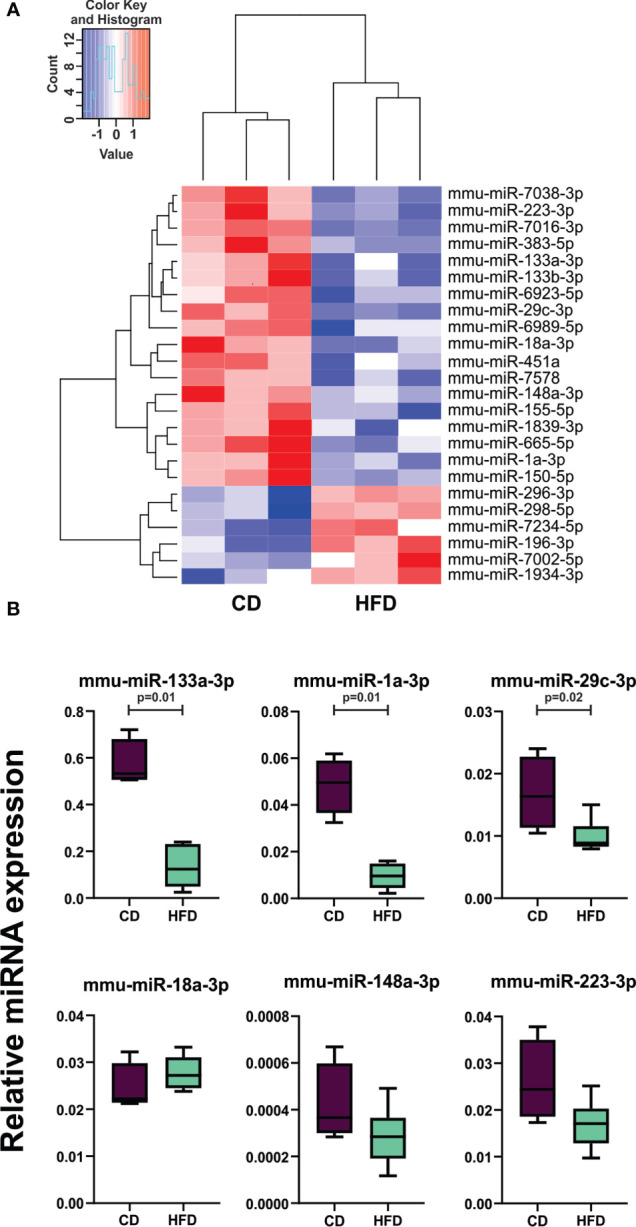
HFD regulates the expression of miRNAs in TRAMP-C1 tumors from mice. **(A)** Heat map representation for miRNA expression in CD and HFD allografts. The heat map was generated based on differentially expressed miRNAs with a FDR <0.05 in TRAMP-C1 tumors developed in HFD compared to CD fed mice. **(B)** Stem-loop RT-qPCR from TRAMP-C1 allografts obtained from CD- or HFD-fed C57BL/6J mice using specific primers for the indicated miRNAs is shown. Data were normalized to mmu-miR-103a-3p and miR-191-5p and control. Statistical analysis was performed using Mann-Whitney test. CD, control diet; HFD, high-fat diet.

Previously, we reported that HFD significantly increased prostate tumor growth, oncogenes and oncomiRs expression in male mice (4–7). In this work, we investigated the impact of HFD on TRAMP-C1 tumor growth developed in C57BL/6J male mice (**Supplemental Figure 1A**). In addition, miRNA expression profile from these tumors was determined using a high-throughput platform. GeneChiP miRNA 4.0 Affymetrix was hybridized with total RNA isolated from CD or HFD TRAMP-C1 tumors. After data normalization, we selected differentially expressed miRNAs with a FDR<0.05 and identified 18 down- and 6 up-regulated miRNAs in HFD tumors compared to CD group (**Figure 2A** and **Table S1**). Since most of the reported works study the role of oncomiRs, in this work we focused on the role of tumor suppressor microRNAs (tsmiRs) in prostate cancer associated to HFD. To validate these results, the expression levels of 6 down-regulated selected miRNAs (mmu-miR-133a-3p, 1a-3p, 29c-3p, 18a-3p, 148a-3p and 223-3p) in TRAMP-C1 tumors from HFD and CD mice were evaluated by RT-qPCR. The results showed a significant decrease in the expression of mmu-miR-133a-3p, 1a-3p and 29c-3p in HFD-tumor compared to CD mice (**Figure 2B**). No changes were detected in mmu-miR-18a-3p, 148a-3p and 223-3p expression levels (**Figure 2B**).

We further determined the expression profile of the selected miRNAs in mice bloodstream. We found that mmu-miR-1a-3p was significantly down-regulated in plasma from HFD mice compared to CD fed animals (**Supplemental Figure 2A**). Mmu-miR-133a-3p, miR-29c-3p, miR-18a-3p and miR-223-3p showed no changes in the levels of their plasma circulation (**Supplemental Figure 2A**).

### Hsa-miR-133a-3p and miR-1-3p are decreased in PCa, while miR-133a-3p is further down-regulated in metastatic PCa tumors

**Figure 3:**
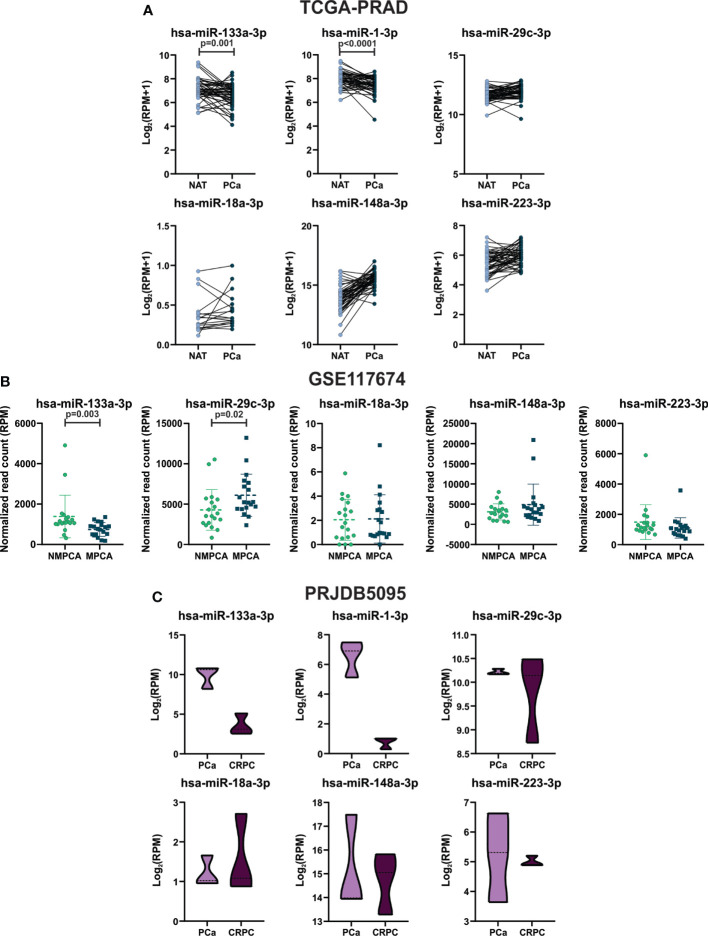
Hsa-miR-133a-3p and miR-1-3p expression in tumor tissue of PCa patients. **(A)** TCGA-PRAD: expression levels of hsa-miR-133a-3p, miR-1-3p, miR-29c-3p, miR-18a-3p, miR-148a-3p and miR-223-3p in prostate primary solid tumor and NAT. Log2 (RPM+1) are graphed. Data were analyzed using paired T-test or Wilcoxon test when appropriate. **(B)** GSE117674: expression levels of hsa-miR-133a-3p, miR-29c-3p, miR-18a-3p, miR-148a-3p and miR-223-3p in NMPCA (non-metastatic PCa) and MPCA (metastatic PCa) tumors. Normalized read count (RPM) values are graphed. Data were analyzed using T-test or Mann-Whitney test when appropriate. **(C)** PRJDB5095: expression levels of hsa-miR-133a-3p, miR-1-3p, miR-29c-3p, miR-18a-3p, miR-148a-3p and miR-223-3p in tumors from patients with CRPC and tumor from PCa patients. Log2(RPM) values are graphed. Data were analyzed using Mann-Whitney test.

To further evaluate the expression profile of the selected miRNAs between CD and HFD tumors in prostate tumors from patients, we performed a bioinformatic analysis using the TCGA-PRAD, GSE117674 and PRJDB5095 datasets. First, we analyzed their expression in prostate primary tumors in comparison to NAT. As shown in **Figure 3A**, hsa-miR-133a-3p and 1a-3p were significantly decreased in PCa compared to NAT (TCGA-PRAD cohort). No significant differences were found regarding the expression of hsa-miR-29c-3p, miR-18a-3p, miR-148a-3p and miR-223-3p.

Since miRNA expression can be altered during prostate cancer progression, the expression profile of the selected miRNAs was also evaluated in metastatic prostate cancer (MPCA) and tumors derived from patients without metastasis. Thus, we explored the miRNome sequencing data from Nam´s study (GSE117674) (17). Hsa-miR-133a-3p was found decreased in tumors from metastatic patients (after surgery) compared to tumors from non-metastatic patients, while hsa-miR-29c-3p was found increased in MPCA (**Figure 3B**). There were no differences in the expression of hsa-miR-18a-3p, miR-148a-3p and miR-223-3p, while hsa-miR-1-3p was not detected in this cohort of patients (**Figure 3B**).

Additionally, we used the miTED tool to evaluate the expression of the miRNAs in tumors from patients with castration-resistant prostate cancer (CRPC). Hsa-miR-133a-3p and hsa-miR-1-3p showed a non-significant diminution in tumors from patients with CRPC compared to PCa (**Figure 3C**). It is important to mention that the number of cases assessed was low (n=3), therefore, in the future, it is necessary to increase the number of patients in order to find statistically significant differences between the groups.

Overall, results suggest that hsa-miR-133a-3p and miR-1-3p might act as tumor suppressors in PCa.

As mentioned above, mmu-miR-1a-3p was found to be decreased in plasma from HFD fed mice (**Supplemental Figure 2A**). In addition, these mice showed elevated fasting blood glucose and cholesterol levels (**Supplemental Figure 1C**), but no changes were found in their body weight (**Supplemental Figure 1B**). Therefore, we investigated the relationship between hyperglycemia and the levels of these miRNAs in plasma. We analyzed the levels of hsa-miR-133a-3p, miR-1-3p, miR-29c-3p, miR-18a-3p, miR-148a-3p and miR-223-3p in blood samples of patients with type 2 diabetes (T2DM) (n=6) and healthy donors (HD) (n=4) (GSE27645). No differences were found in circulating levels of the 6 miRNAs (**Supplemental Figure 2B**).

### Hsa-miR-133a-3p and miR-1-3p share several target genes

**Figure 4:**
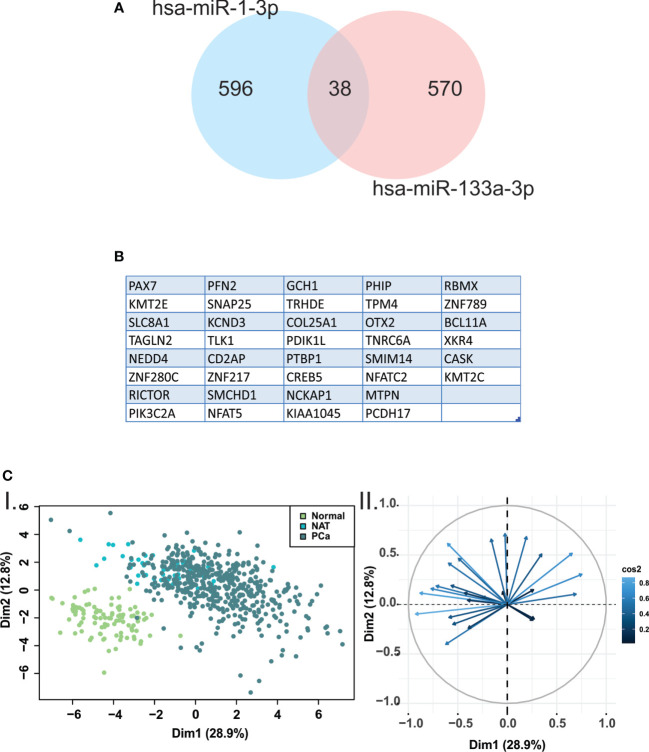
Principal components analysis revealed marked differences in hsa-miR-133a-3p and miR-1-3p target genes expression between normal prostate and PCA samples. **(A)** Venn diagrams were performed using predicted target genes from hsa-miR-133a-3p and miR-1-3p. **(B)** The 38 target genes shared between hsa-miR-133a-3p and miR-1-3p are listed. **(C)** I. Scatterplot of the two principal components of principal component analysis (PCA) from the 38 common target genes expression data. The green, light blue and blue circles represent normal prostate, normal adjacent tissue (NAT) and PCa samples, respectively. II. Biplot representing the common genes and their relevance in each dimension of the plot.

Due to no validated target gene data was found for the miRNAs included in this study, we obtained a list of predicted target genes for hsa-miR-133a-3p and miR-1-3p, using microT-CDS resource. To identify common target genes, we used Venn diagrams (**Figure 4A**). We found 608 target genes for hsa-miR-133a-3p, while 634 target genes for hsa-miR-1-3p. There were 38 target genes in common between hsa-mR-133a-3p and miR-1-3p (**Figures 4A, B**). Additionally, to determine the relevance of down-regulated miRNAs-target genes in human samples, we performed a PCA of the 38 common target genes using the normalized expression data from normal prostate (GTEX), NAT and prostate tumors samples (TCGA-PRAD). The 2-dimensional scatterplot of the first two principal components revealed marked differences in overall gene expression between normal prostate and PCa samples (**Figure 4C**).

### Down-regulated miRNAs by HFD modulate cancer related pathways

**Figure 5:**
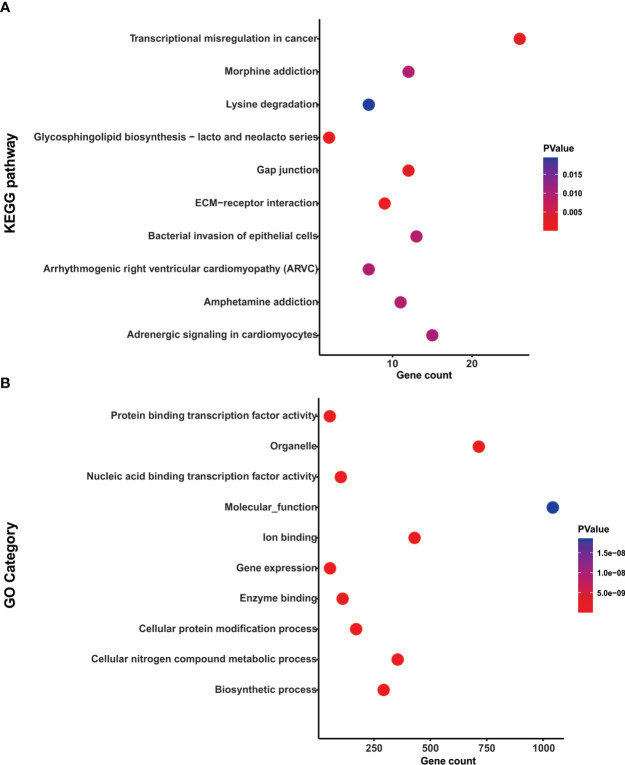
Functional enrichment analysis of hsa-miR-133a-3p and miR-1-3p. **(A)** Dotplot representation of the top 10 significant KEGG pathways associated with the target genes of hsa-miR-133a-3p and miR-1-3p. **(B)** Dotplot representation of the top 10 significant GO category associated with the target genes of hsa-miR-133a-3p and miR-1-3p.

To explore the functional role of the selected miRNAs and pathways, we used DIANA-miRPath v3 tool (http://snf-515788.vm.okeanos.grnet.gr/). With all the predicted target genes, we performed a KEGG pathway enrichment analysis using a p-value <0.05. This analysis revealed that target genes of hsa-miR-133a-3p and miR-1-3p were associated with processes such as Transcriptional misregulation in cancer and ECM−receptor interaction (**Figure 5A** and **Table S4**).

Also, using DIANA-miRPath v3, a GO analysis was performed at three levels: biological processes, cellular components and molecular function. The top 10 of the statistically significant terms (p-value <0.05) are shown in **Figure 5B**. Target genes hsa-miR-133a-3p and miR-1-3p were enriched in processes associated with Nucleic acid binding transcription factor activity, Gene expression and Protein binding transcription factor activity (**Figure 5B** and **Table S5**). Therefore, hsa-miR-133a-3p and miR-1-3p modulated specific cancer related pathways.

### GOLPH3 and JUP are increased in primary prostate tumors from patients

**Figure 6:**
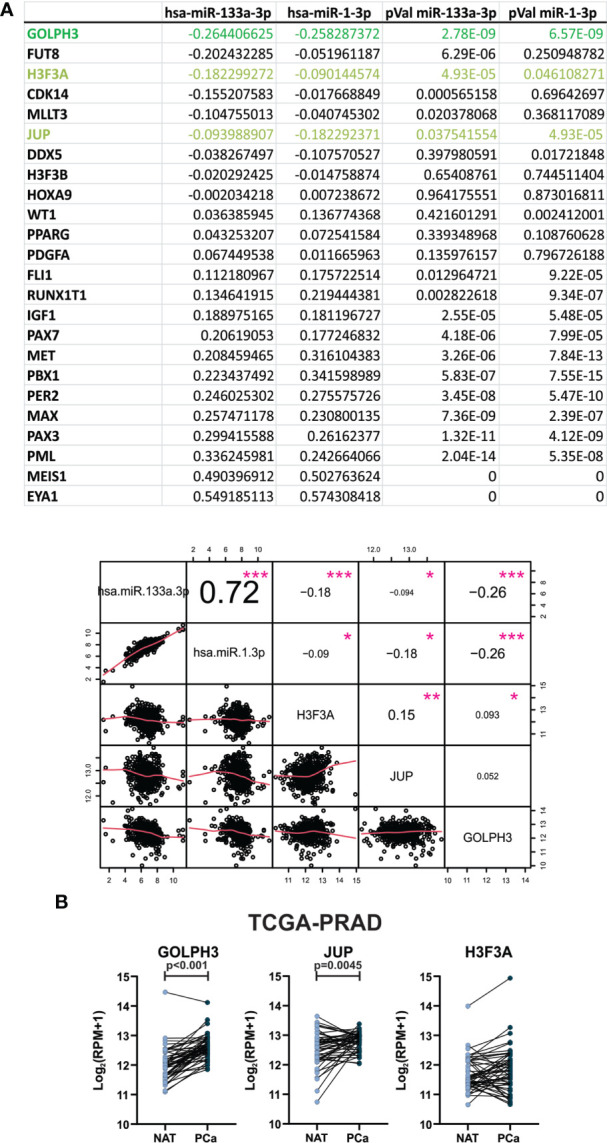
GOLPH3 and JUP expression are increased in primary prostate tumors from patients. **(A)** Spearman correlation between hsa-miR-133a-3p and miR-1-3p expression in PCa tissue from TCGA-PRAD dataset, and the selected target genes is plotted. Spearman correlation value and p-values are indicated in the table and in the graph as numbers and pink asterisks respectively. **(B)** GOLPH3 and JUP expression levels in paired PCa tissues compared with NAT by analyzing the PCa mRNA sequencing dataset from TCGA-PRAD (NAT, n = 52; PCa, n = 52). Read per millions values are graphed. Data were analyzed using paired T-test or Wilcoxon test when appropriate. *p-value < 0.05, **p-value < 0.01, ***p-value < 0.001.

In order to find relevant target genes for the 2 selected miRNAs, we performed a correlation matrix, using the expression data of hsa-miR-133a-3p and miR-1-3p and the 26 target genes involved in the processes Transcriptional misregulation in cancer (**Table S4**) of prostate tumors from patients available in TCGA-PRAD (UCSC Xena). From this analysis, we selected the target genes that showed a negative Spearman correlation coefficient rho and a p-Value <0.05 with the 2 miRNAs (**Figure 6A**). We found 3 target genes (GOLPH3, H3F3A and JUP) with a significant negative correlation with the selected miRNAs (**Figure 6A**). Also, hsa-miR-133a-3p and miR-1-3p showed a strong positive correlation between them with a significant p-value (**Figure 6A**). Then, we analyzed the expression pattern of the three target genes in prostate tumors and normal samples from TCGA-PRAD. As shown in **Figure 6B**, we found that GOLPH3 and JUP expression was significantly increased in primary prostate tumors compared to NAT. There were no differences in the expression of H3F3A.

### Hsa-miR-133a-3p, miR-1-3p, GOLPH3 and JUP combination results in a good biomarker to distinguish between PCa from non-PCa patients

**Figure 7:**
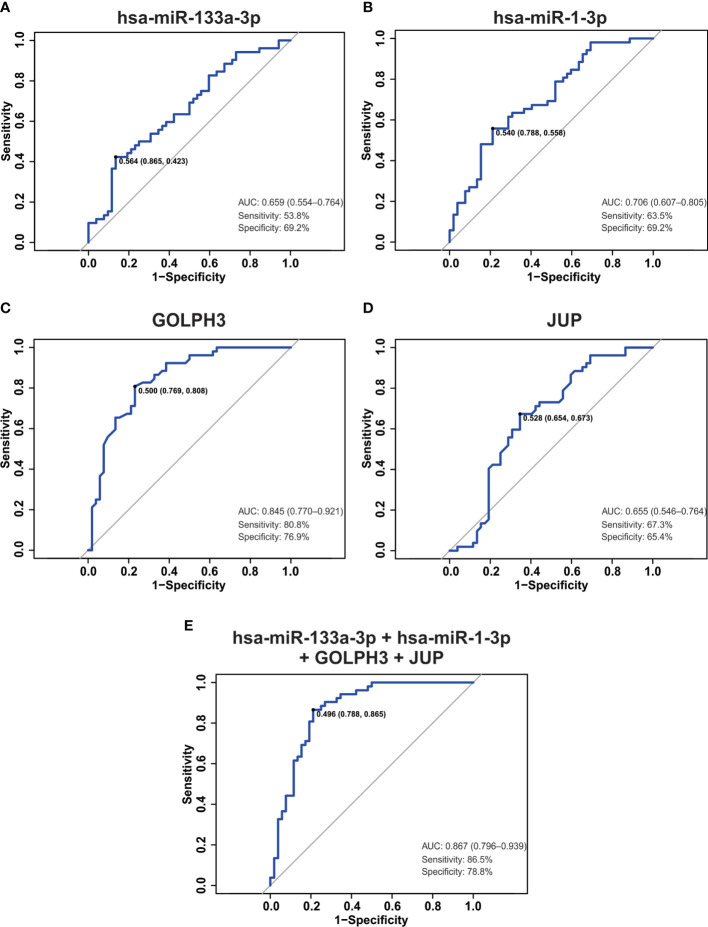
ROC analysis in tumor dataset. **(A–E)** ROC curve, the AUC, sensitivity, specificity and the optimal point were plotted and calculated for hsa-miR-133a-3p **(A)**, miR-1-3p **(B)**, GOLPH3 **(C)**, JUP **(D)** and the combination of them **(E)**.

Up to date, digital rectal examination (DRE) and serum prostate-specific antigen (PSA) monitoring are the standard methods of PCa screening (18). However, PSA is organ- but not tumor-specific biomarker with low specificity and high false-positive rate in patients with benign prostatic hyperplasia (BPH) (18, 19).Therefore, we studied weather hsa-miR-133a-3p, miR-1-3p, GOLPH3, JUP or a combination of them, resulted in a good biomarker to distinguish between PCa and non-PCa patients. To do this, we performed a receiver-operating characteristic (ROC) analysis. As shown in **Figures 7A, B, D** poor predictive power to discriminate between tumor tissue and NAT was obtained when we calculated the area under the curve (AUC) for hsa-miR-133a-3p, miR-1-3p and JUP in (0.659, 0.706 and 0.655, respectively). AUC for GOLPH3 was 0.845 (**Figure 7C**), which shows that these gene is useful to distinguish between tumor tissue and NAT. However, a gene panel might be used in the clinic instead of a single diagnostic biomarker. For this reason, we analyzed a combination of genes and miRNAs (hsa-miR-133a-3p, miR-1-3p, GOLPH3 plus JUP) as a possible biomarker panel to distinguish between tumor tissue and NAT. It was shown that this combination improved the performance of GOLPH3, obtaining an AUC of 0.867, 86.5% sensitivity and 78.8% specificity (**Figure 7E**). Therefore, this combination resulted in a good diagnostic biomarker panel able to discriminate between tumor tissue and non-tumor tissue.

### Hsa-miR-1-2/miR-133a-1 promoter hypermethylation is increased in PCa and negatively correlated with miRNA expression

**Figure 8:**
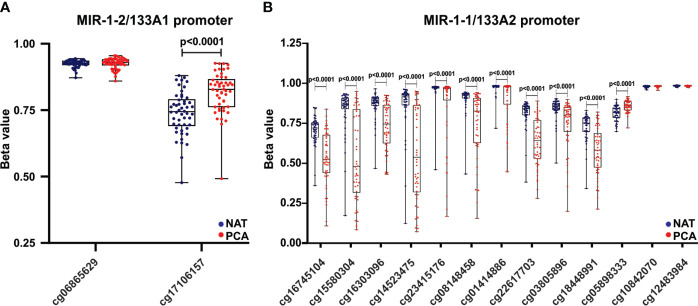
Hsa-miR-1-2/miR-133a-1 promoter hypermethylation is increased in PCa and negatively correlated with miRNA expression. The methylation levels of hsa-miR-1-2/133a1 **(A)** and hsa-miR-1-1/133a2 **(B)** promoter regions were estimated from the TCGA-PRAD dataset in 52 paired PCa tissues compared to NAT. Beta values are graphed. Data were analyzed using paired t-test.

The hsa-miR-1/133 family is located at three different loci (as clustered miRNAs) at chromosomes 18q11.2 (miR-1-2/miR-133a-1), 20q13.33 (miR-1-1/miR-133a-2), and 6p12.2 (miR-206/miR-133b) (20). Here, we analyzed the methylation status at miR-1-2/miR-133a-1 and miR-1-1/miR-133a-2 promoters. Based upon the Ensembl and UCSC Xena resources, two and thirteen methylation probes were identified targeting the hsa-miR-1-2/miR-133a-1 and miR-1-1/miR-133a-2 promoters, respectively (**Figure 8**). We compared the methylation status of two cluster promoters (miR-1-2/miR-133a-1 and miR-1-1/miR-133a-2) in 52 paired PCa and NAT samples. As shown in **Figure 8A**, one probe (cg17106157) targeting miR-1-2/133a-1 showed significantly higher beta values in PCa compared to NAT. Also, ten and one probes targeting miR-1-1/133a-2 promoter showed lower and higher beta values in PCa vs NAT respectively (**Figure 8B**). Then, a correlation analysis between promoter methylation and mature miRNA expression levels was performed in 497 PCa samples. As presented in **Table S3**, Spearman correlation analysis showed that hsa-miR-133a-3p and hsa-miR-1-3p expression negatively correlated with cg17106157 (chromosome 18) probe and with cg05898333 (chromosome 20) probe. Moreover, hsa-miR-133a-3p and hsa-miR-1-3p expression positively correlated with cg15580304, cg14523475, cg08148458 and cg22617703 probes targeting the miR-1-1/miR-133a-2 promoter. Therefore, the decreased expression observed in hsa-miR-1-3p/miR-133a-3p in prostate tumors might be due to a hsa-miR-1-2/miR-133a-1 cluster promoter hypermethylation.

## Discussion

Here we demonstrated that HFD markedly reduces the expression of potential tsmiRs in TRAMP-C1 tumors developed as allograft in C57BL/6J mice. Target genes modulated by these tsmiRs regulate processes mainly associated to cancer related pathways. Among these, mmu-miR-133a-3p and miR-1a-3p were dramatically diminished in tumors by HFD. Additionally, human orthologous miRNAs were significantly down-regulated in human prostate tumors compared to NAT and normal prostates. Moreover, we found that hsa-miR-133a-3p and miR-1-3p were significantly decreased in prostate tumors of metastatic patients compared to tumors of non-metastatic patients.

The hsa-miR-1, -133 and -206 family are located at three different loci at chromosomes 20q13.33 (miR-1-1/miR-133a-2), 18q11.2 (miR-1-2/miR-133a-1) and 6p12.2 (miR-206/miR-133b) (20). The mature miR-133 isomers (A and B) are highly similar, differing only at the 3′-terminal base, with miR-133a-1/2 terminating G-3′ and miR-133b with A-3′, respectively (21). Due to their close locations at distinct loci, miR-1/133a, miR-206/133b are constituted as clustered miRNAs (21). Recent studies showed miR-1, -133 and -206 family deregulation in cancer, in which typically they function as tumor suppressors (22, 23).

Hsa-miR-133a-3p is probably the most studied miRNA of this family and has been extensively reported as down-regulated in several types of cancer and predicted a poor prognosis (24–29). In PCa, a low expression of hsa-miR-133a-3p has been associated with the recurrence and distant metastasis of PCa (30–33). Likewise, a recent study from Tang et al. demonstrated that hsa-miR-133a-3p expression is reduced in PCa tissues compared with the NAT and benign prostate lesion tissues, particularly in bone metastatic PCa tissues. Also, low expression of miR-133a-3p is significantly correlated with advanced clinicopathological characteristics and shorter bone metastasis-free survival in PCa patients (34).

As referred to hsa-miR-1, different studies demonstrated that is the most down-regulated miRNA in PCa compared to non-cancerous prostate tissues and significantly decreased in recurrent PCa specimens in comparison to non-recurrent PCa samples (35–37). MiR-1 is further down-regulated in cancer progression and alone can predict disease recurrence (36). Also, miR-1 has sufficient power to distinguish recurrent PCa specimens from non-recurrent (35, 36, 38). Furthermore, it’s has been reported to target the oncogenic function of purine nucleoside phosphorylase (PNP) in PCa (38). Thus, hsa-miR-1 may exert similar tumor suppressor activities and coordinately regulate the expression of oncogenes controlling PCa initiation and progression.

Based on our and other groups’ findings, we propose hsa-miR-133a-3p and miR-1-3p as promising miRNAs to be studied as potential biomarkers for PCa diagnosis and prognosis.

In this work, we also aim to find relevant target genes for hsa-miR-133a-3p and miR-1-3p. We performed a correlation analysis using data from PCa patients available in public algorithms. This analysis allowed us to find three relevant target genes for the two miRNAs with a significant negative correlation. Only two of the three target genes, GOLPH and JUP were significantly increased in primary prostate tumors compared to NAT. In addition, ROC analysis showed that the combination of hsa-miR-133a-3p, miR-1-3p, GOLPH3 and JUP is a promising panel biomarker to distinguish between PCa and NAT.

In addition, we performed a bioinformatic analysis to search experimentally validated target genes of hsa-miR-133a-3p and miR-1-3p, involved in the androgen receptor (AR) pathway. Although the AR gene is not a target of these miRNAs, we found that several AR co-regulator genes (MNAT1, GTF2H1, RAN) were direct targets of hsa-miR-133a-3p and miR-1-3p. In turn, we found that genes involved in the MAPK signaling pathway (F2RL1, HRAS), a major oncogenic and AR crosstalk pathway in PCa, were experimentally validated targets of hsa-miR-1-3p. We suggest that hsa-miR-133a-3p and miR-1-3p act as tumor suppressor miRNAs in PCa because they interfere in the AR signaling pathway, the central pathway for PCa initiation and growth. In particular, these miRNAs would be involved in the castration resistance process, since they have several AR co-regulators as direct targets. Furthermore, both miRNAs showed a tendency to be decreased in more aggressive prostate tumors. Therefore, hsa-miR-133a-3p and miR-1-3p emerge as potential tsmiRs whose attenuation would increase PCa aggressiveness.

MiRNAs may be epigenetically silenced by DNA methylation of their encoding genes (9). DNA hypermethylation was found to down-regulate several tsmiRNAs, whereas DNA hypomethylation was reported to up-regulate oncomiRs (39). In this study, we analyzed the methylation status at miR-1-2/miR-133a-1 and miR-1-1/miR-133a-2 promoters in PCa and normal samples and examined the correlation between promoter methylation and mature miRNA expression. This bioinformatics analysis suggests that hypermethylation of hsa-miR-1-2/miR-133a-1 cluster promoter might decrease hsa-miR-1-3p/miR-133a-3p expression in prostate tumors. Therefore, epigenetic repression of the hsa-miR-1-2/miR-133a-1 cluster may play a critical role in PCa aggressiveness by activating GOLPH3 and JUP.

Golgi phosphoprotein 3 (GOLPH3) is an oncogene involved in the development of carcinoma in a number of organs and a candidate metastasis gene in human cancer (40, 41).Different studies have investigated the role of GOLPH3 in PCa. Independent reports demonstrated that GOLPH3 overexpression in PCa tissues is linked to higher Gleason grade, advanced pathological tumor stage, the presence of metastasis, worst overall survival and the state of the lymph nodes (40, 42, 43). Overexpression of GOLPH3 is associated with the transition of hormone sensitive to hormone refractory PCa (43). Li and Guo reported that GOLPH3 silencing inhibited cell proliferation and arrested the cell cycle at the G2/M phase (44). Also, GOLPH3 silencing activated P21 expression but suppressed the expression of CDK1/2 and cyclinB1 protein together with the phosphorylation of AKT and mTOR (44). Further revealed that silencing of GOLPH3 reduced cell migration and invasion ability (41). Finally, *in vitro* studies demonstrated that GOLPH3 regulates cell size, enhances growth factor-induced mTOR signaling in human cancer cells and modulates the response to rapamycin (45).

The role of junction plakoglobin (JUP) during cancer progression is still controversial. Spethmann et al., demonstrated that the opposing biological roles of JUP were reflected by antagonistic prognostic effects in different molecular subtypes. High expression of JUP was associated with adverse tumor stage, high Gleason grade lymph node metastases in a subset of PCa patients without TMPRSS2:ERG fusion (46). Also, Overexpression of JUP was linked to strong androgen receptor expression, high cell proliferation, and PTEN and FOXP1 deletion (46). On the other hand, it was found that SOX4 interacts with JUP in a Wnt-dependent manner in LNCaP cells and this complex may inhibit Wnt signaling (47).

Therefore, based on our and previous studies, GOLPH3 and JUP have a critical role in PCa pathogenesis and progression.

In conclusion, our results demonstrated that HFD dramatically reduces the expression of tsmiRs in androgen-sensitive prostate tumors. Additionally, the expression of hsa-miR-133a-3p and miR-1-3p negatively correlates with GOLPH3 and JUP, two PCa driver oncogenes.

Besides, hsa-miR-133a-3p and miR-1-3p are epigenetically silenced by promoter hypermethylation and functions as tsmiRs in PCa.

Although evaluations of the two miRNAs and their target genes expression in larger populations are still needed, our results indicate that hsa-miR-133a-3p, miR-1-3p, GOLPH3 and JUP are functional drivers of PCa and may be their combination is a promising diagnostic biomarker panel for prostate cancer.

In conclusion, our results demonstrated that HFD modulates the expression of a substantial number of miRNAs in PCa. Attenuation of hsa-miR-133a-3p and miR-1-3p expression by promoter methylation in prostate tumors may enhance PCa development, in part by targeting GOLPH3 and JUP. Hsa-miR-133a-3p, miR-1-3p, GOLPH3 and JUP are functional drivers of PCa and may be their combination is a promising diagnostic biomarker panel for prostate cancer.

## Data availability statement

The datasets presented in this study can be found in online repositories. The names of the repository/repositories and accession number(s) can be found below: https://www.ncbi.nlm.nih.gov/geo/query/acc.cgi?acc=GSE181350.

## Ethics statement

The animal study was reviewed and approved by Comité Institucional para el Cuidado y Uso de Animales de Laboratorio-IBYME.

## Author contributions

RD and AD designed the study. RD and CM carried out the experiments with contributions of PF, KDG and JM. RD and EL performed bioinformatics analyses. RD, CM, EL, and ADS interpreted the data. RD, CM and ADS wrote the manuscript with contributions of KG and approval from all authors. All authors contributed to the article and approved the submitted version.

## Funding

This research was supported by the Argentinean Agency of Science and Technology (ANPCyT PICT 2014-324; PICT 2015-1345, PICT 2018-1304, PICT START UP-2019-21), National Cancer Institute (Argentina) (INC 2020) and Williams Foundation (Argentina). This work was part of Ph.D. thesis of RD supported by the CONICET fellowship from Argentina.

## Acknowledgments

The authors thank National Cancer Institute (Argentina), Williams Foundation (Argentina) and Argentinean Agency of Science and Technology for their support.

## Conflict of interest

The authors declare that the research was conducted in the absence of any commercial or financial relationships that could be construed as a potential conflict of interest.

## Publisher’s note

All claims expressed in this article are solely those of the authors and do not necessarily represent those of their affiliated organizations, or those of the publisher, the editors and the reviewers. Any product that may be evaluated in this article, or claim that may be made by its manufacturer, is not guaranteed or endorsed by the publisher.

## Abbreviations

AUC, area under the curve; BPH, benign prostatic hyperplasia; CD, control diet; CRPC, castration-resistant prostate cancer; DRE, digital rectal examination; GOLPH3, golgi phosphoprotein 3; HD, healthy donor; HFD, high fat diet; JUP, junction plakoglobin; MeS, metabolic syndrome; miRNA, microRNA; MPCA, metastatic prostate cancer; NAT, normal adjacent tissue; NMPCA, non-metastatic prostate cancer; OncomiR, oncogenic miRNA; PCa, prostate cancer; PSA, prostate-specific antigen; ROC, receiver-operating characteristic; T2DM, type 2 diabetes; TsmiR, Tumor suppressor miRNA.
