# Supplementary material for: Hsa-miR-133a-3p, miR-1-3p, GOLPH3 and JUP combination results in a good biomarker to distinguish between prostate cancer and non-prostate cancer patients
Source: Front Oncol. 2022 Oct 26;12:997457. doi: 10.3389/fonc.2022.997457 (PMC9641240; doi:10.3389/fonc.2022.997457)
Supplement: Supplementary file 1 [file DataSheet_1.pdf]

Table S1. List of deregulated miRNAs by HFD in TRAMP-C1 tumors

Probe ID	miRNA	log2FoldChange	FDR
MIMAT0000665_st	mmu-miR-223-3p	- 0.95	0.04352941
MI0001730_x_st	mmu-miR-451a	- 1.06	0.03642857
MIMAT0000748_st	mmu-miR-383-5p	- 1.08	0.046842106
MIMAT0027880_st	mmu-miR-6989-5p	- 1.12	0.036666665
MIMAT0000165_st	mmu-miR-155-5p	- 1.12	0.03642857
MIMAT0017238_st	mmu-miR-665-5p	- 1.15	0.04222222
MIMAT0009457_st	mmu-miR-1839-3p	- 1.21	0.038125
MIMAT0027746_st	mmu-miR-6923-5p	- 1.23	0.016363636
MIMAT0004626_st	mmu-miR-18a-3p	- 1.26	0.01
MIMAT0027937_st	mmu-miR-7016-3p	- 1.29	0.017
MIMAT0000516_st	mmu-miR-148a-3p	- 1.31	0.03642857
MIMAT0000536_st	mmu-miR-29c-3p	- 1.39	0.0057142857
MIMAT0000160_st	mmu-miR-150-5p	- 1.49	0.0057142857
MI0024714_st	mmu-miR-7578	- 1.53	0.006
MIMAT0000123_st	mmu-miR-1a-3p	- 1.54	0.01
MIMAT0027981_st	mmu-miR-7038-3p	- 1.57	0.006
MIMAT0000769_st	mmu-miR-133b-3p	- 3.05	0
MIMAT0000145_st	mmu-miR-133a-3p	- 3.45	0
MIMAT0000376_st	mmu-miR-298-5p	- 2.12	0
MIMAT0028436_st	mmu-miR-7234-5p	1.63	0
MIMAT0004576_st	mmu-miR-296-3p	1.35	0.036
MIMAT0017341_st	mmu-miR-1934-3p	1.27	0.026666667
MIMAT0027906_st	mmu-miR-7002-5p	1.21	0.0225
MIMAT0017170_st	mmu-miR-196b-3p	1.13	0.036666665

Table S2. Primer sequences used for stem-loop RT-qPCR.

Primer	Sequence (5'-3')	T ann (°C)
RT-Stem-loop-Rv	TGGTGCAGGGTCCGAGGTATT	-
RT-mmu-miR-133a-3p-STEM	GTCTCCTCTGGTGCAGGGTCCGAGGTATTCGCACCAGAGGAGACCAGCTG	-
RT-mmu-miR-133a-3p Fw	CGGGCGGTTTGGTCCCCTTCAAC	65
RT-mmu-miR-1a-3p-STEM	GTCTCCTCTGGTGCAGGGTCCGAGGTATTCGCACCAGAGGAGACATACAT	-
RT-mmu-miR-1a-3p Fw	CGGGCGGTGGAATGTAAAGAAG	67
RT-mmu-miR-29c-3p-STEM	GTCTCCTCTGGTGCAGGGTCCGAGGTATTCGCACCAGAGGAGACTAACCG	-
RT-mmu-miR-29c-3p Fw	GCGGCGGTAGCACCATTTGAA	62
RT-mmu-miR-18a-3p-STEM	GTCTCCTCTGGTGCAGGGTCCGAGGTATTCGCACCAGAGGAGACCAGAAG	-
RT-mmu-miR-18a-3p Fw	GTCTACTGCCCTAAGTGCTC	65
RT-mmu-miR-148a-3p-STEM	GTCTCCTCTGGTGCAGGGTCCGAGGTATTCGCACCAGAGGAGACACAAAG	-
RT-mmu-miR-148a-3p Fw	GGTATTCAGTGCACTACAG	62
RT-mmu-miR-223-3p-STEM	GTCTCCTCTGGTGCAGGGTCCGAGGTATTCGCACCAGAGGAGACTGGGGT	-
RT-mmu-miR-223-3p Fw	GGGCGGCTGTCAGTTTGTC	65
RT-mmu-miR-191-5p-STEM	GTCTCCTCTGGTGCAGGGTCCGAGGTATTCGCACCAGAGGAGACCAGCTG	-
RT-mmu-miR-191-5p Fw	GCGGCAACGGAATCCCAAAAG	70
RT-mmu-miR-103a-3p-STEM	GTCTCCTCTGGTGCAGGGTCCGAGGTATTCGCACCAGAGGAGACTCATAG	-
RT-mmu-miR-103a-3p Fw	GGGGAGCAGCATTGTACAGGG	67
RT-miR-Cel39-STEM	GTCTCCTCTGGTGCAGGGTCCGAGGTATTCGCACCAGAGGAGACCAAGCT	-
RT-miR-Cel39 Fw	CGGGGTCACCGGGTGTAAATC	65

Table S3. Spearman correlation analysis of methylation probes's values with miRNA expression

Probe	Probe genomic location	miRNA genomic location	Spearman coefficient	P value	mature miRNA
cg06865629	18: 19,409,163-19,409,165	**MIR133A1** 18:19,405,659-19,405,746	0.01791	0.6921	hsa-miR-133a-3p
**cg17106157**	**18: 19,410,149-19,410,151**		**-0.2188**	**<0.0001**	
cg06865629	18: 19,409,163-19,409,165	**MIR1-2** 18:19,408,965-19,409,049	0.04883	0.2802	hsa-miR-1-3p
**cg17106157**	**18: 19,410,149-19,410,151**		**-0.2621**	**<0.0001**	
cg16745104	20: 62,550,414-62,550,416	**MIR133A2** 20: 62,564,912-62,565,013	0.01571	0.7283	hsa-miR-133a-3p
**cg15580304**	**20: 62,549,384-62,549,386**		**0.1713**	**0.0002**	
**cg16303096**	**20: 62,551,783-62,551,785**		**0.1226**	**0.0065**	
**cg14523475**	**20: 62,551,912-62,551,914**		**0.194**	**<0.0001**	
cg23415176	20: 62,550,739-62,550,741		0.03639	0.4211	
**cg08148458**	**20: 62,551,653-62,551,655**		**0.2196**	**<0.0001**	
cg01414886	20: 62,549,358-62,549,360		0.05243	0.2462	
**cg22617703**	**20: 62,550,733-62,550,735**		**0.1317**	**0.0035**	
cg03805896	20: 62,545,439-62,545,441		0.008004	0.8596	
**cg18448991**	**20: 62,550,713-62,550,715**		**0.1049**	**0.0201**	
**cg05898333**	**20: 62,551,361-62,551,363**		**-0.2227**	**<0.0001**	
cg10842070	20: 62,550,466-62,550,468		0.04968	0.2719	
**cg12483984**	**20: 62,551,208-62,551,210**		**0.09832**	**0.0294**	
cg16745104	20: 62,550,414-62,550,416	**MIR1-1** 20: 62,554,306-62,554,376	0.01178	0.7946	hsa-miR-1-3p
**cg15580304**	**20: 62,549,384-62,549,386**		**0.1704**	**0.0002**	
cg16303096	20: 62,551,783-62,551,785		0.07435	0.0999	
**cg14523475**	**20: 62,551,912-62,551,914**		**0.1922**	**<0.0001**	
cg23415176	20: 62,550,739-62,550,741		-0.01443	0.7497	
**cg08148458**	**20: 62,551,653-62,551,655**		**0.2026**	**<0.0001**	
cg01414886	20: 62,549,358-62,549,360		-0.04484	0.3214	
**cg22617703**	**20: 62,550,733-62,550,735**		**0.1322**	**0.0033**	
cg03805896	20: 62,545,439-62,545,441		0.01108	0.8065	
cg18448991	20: 62,550,713-62,550,715		0.07972	0.0776	
**cg05898333**	**20: 62,551,361-62,551,363**		**-0.2671**	**<0.0001**	
cg10842070	20: 62,550,466-62,550,468		-0.01417	0.7542	
cg12483984	20: 62,551,208-62,551,210		0.05089	0.2604	

Table S4. 10 top KEGG signaling pathways with a pValue<0.05 for hsa-miR-133a-3p and miR-1-3p target genes

KEGG pathway	PValue	Gene count	miRNAs count
Glycosphingolipid biosynthesis - lacto and neolacto series	3.07E-06	2	1
ECM-receptor interaction	7.12E-06	9	2
Gap junction	2.82E-03	12	2
Transcriptional misregulation in cancer	2.82E-03	26	2
Bacterial invasion of epithelial cells	0.00924611	13	2
Amphetamine addiction	0.00953225	11	2
Morphine addiction	0.00953225	12	2
Arrhythmogenic right ventricular cardiomyopathy (ARVC)	0.00953225	7	2
Adrenergic signaling in cardiomyocytes	0.01052284	15	2
Lysine degradation	0.01947552	7	2

Table S5. 10 top GO category with a pValue<0.05 for hsa-miR-133a-3p and miR-1-3p target genes

GO Category	PValue	Gene count	miRNAs count
Organelle	4.50E-55	715	2
Cellular nitrogen compound metabolic process	9.51E-27	355	2
Ion binding	9.19E-25	430	2
Biosynthetic process	1.92E-17	293	2
Nucleic acid binding transcription factor activity	4.80E-15	103	2
Cellular protein modification process	2.68E-10	171	2
Gene expression	3.19E-10	55	2
Protein binding transcription factor activity	5.05E-10	54	2
Enzyme binding	5.05E-10	111	2
Molecular_function	1.86E-08	1043	2

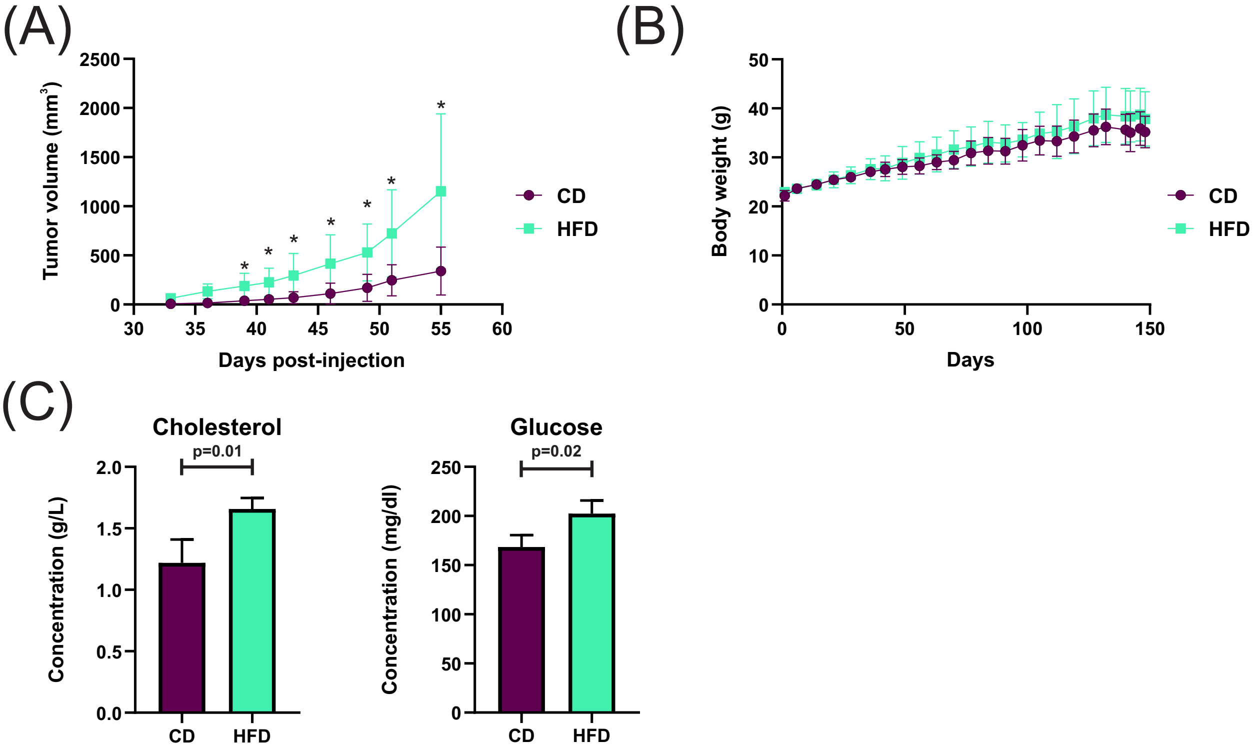

Supplemental Figure 1. PCa and HFD model. Six-week-old C57 BL/6J mice were fed with CD o HFD for the entirety of the experiment. (A) Tumor size was measured three times a week. (B) Body weight was measured once a week and on the 12th week of diet mice were inoculated in the left flank with TRAMP-C1 cells. (C) Cholesterol and fasting blood glucose were measured at 12th week.

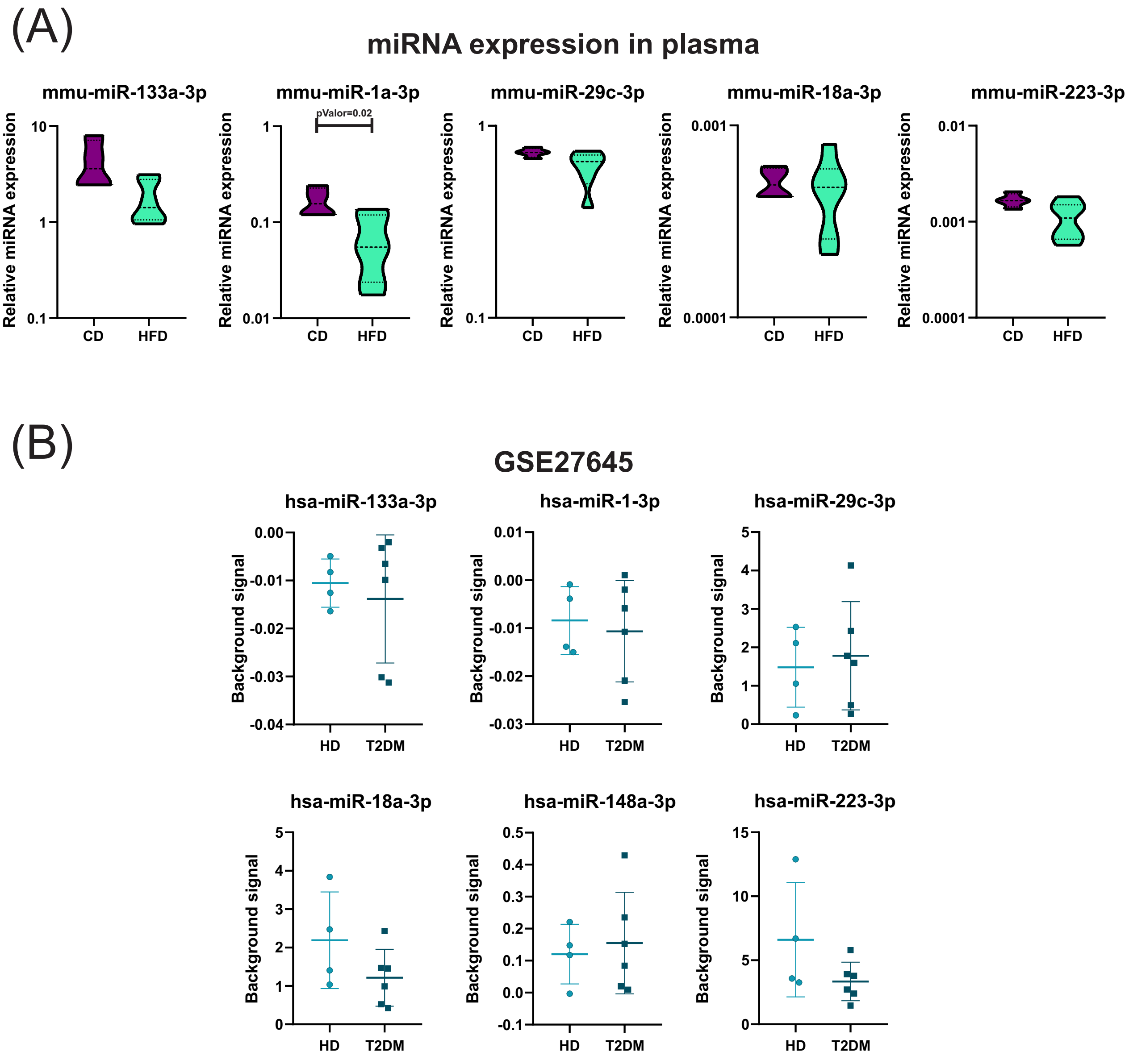

Supplemental Figure 2. Circulation of HFD-down-regulated miRNAs in blood of mice and patients. (A) Mice plasma samples were analyzed by stem-loop RT-qPCR for mmu-miR-133a-3p, miR-1a-3p, miR-29c-3p, miR-18a-3p and miR-223-3p and normalized to spike-in cel-miR-39 synthetic miRNA. 2ΔCt is graphed (ΔCt value = Ctcel-39 - Ctsample). Data were analyzed by Mann-Whitney test. (B) GSE27645: Circulating miRNA expression levels of hsa-miR-133a-3p, miR-1-3p, miR-29c-3p, miR-18a-3p, miR-148a-3p and miR-223-3p in plasma of patients with type 2 diabetes and healthy donor samples. Background signal values are plotted. Data were analyzed by Mann-Whitney test.
